# Supplementary material for: Single-pass transformation of syngas into ethanol with high selectivity by triple tandem catalysis
Source: Nat Commun. 2020 Feb 11;11:827. doi: 10.1038/s41467-020-14672-8 (PMC7012879; doi:10.1038/s41467-020-14672-8)
Supplement: Supplementary file 2 — Supplementary Information [file 41467_2020_14672_MOESM2_ESM.pdf]

Supplementary Information

Single-pass transformation of syngas into ethanol with high selectivity by triple tandem catalysis

Kang et al.

Table of content

Supplementary Figures

Supplementary Figure 1 Thermodynamic analyses.

Supplementary Figure 2 Characterizations of metal oxides.

Supplementary Figure 3 EPR spectra for ZrO_2, $ZnO-ZrO_2$ and $K^+-ZnO-ZrO_2$ samples.

Supplementary Figure 4 Characterizations of H-MOR and H-MOR−DA−12MR zeolites.

Supplementary Figure 5 ^{23}Na MAS NMR spectra for MOR zeolites.

Supplementary Figure 6 ^{27}Al MAS NMR spectra for H-MOR and H-MOR−DA−12MR samples.

Supplementary Figure 7 TEM micrographs with particle size distributions.

Supplementary Figure 8 Dark-field TEM micrographs with line-scan EDS analyses.

Supplementary Figure 9 HRTEM images for Pt/SiC and Pt−Sn/SiC.

Supplementary Figure 10 XPS and CO chemisorption results for Pt/SiC and Pt−Sn/SiC.

Supplementary Figure 11 Effect of temperature on syngas conversion on $K^+-ZnO-ZrO_2$.

Supplementary Figure 12 Reproducibility of syngas conversion.

Supplementary Figure 13 Arrhenius plots.

Supplementary Figure 14 Effects of partial pressures of CO and H_2.

Supplementary Figure 15 Characterizations of used H-MOR and H-MOR−DA−12MR.

Supplementary Tables

Supplementary Table 1 Typical catalysts reported for the conversion of syngas to ethanol.

Supplementary Table 2 Typical catalysts reported for the conversion of syngas to acetic acid.

Supplementary Table 3 Conversion of syngas using combination of $K^+-ZnO-ZrO_2$|H-MOR−DA−12MR|Cu/SiO_2 at different temperatures.

Supplementary Table 4 Effect of Sn content in Pt−Sn/SiC on catalytic performances of $K^+-ZnO-ZrO_2$|H-MOR−DA−12MR|Pt−Sn/SiC for syngas conversion.

Supplementary Table 5 Effect of Pt loading in Pt−Sn/SiC on catalytic performances of K^+−ZnO−ZrO_2|H-MOR−DA−12MR|Pt−Sn/SiC for syngas conversion.

Supplementary Table 6 Effect of ratio of amounts of three catalyst components on catalytic performances of K^+−ZnO−ZrO_2|H-MOR−DA−12MR|Pt−Sn/SiC for syngas conversion.

Supplementary Table 7 Densities of Brønsted acid sites in 8-MR, 12-MR and intersection between 8-MR and 12-MR estimated from ^1H MAS NMR and FT-IR spectra.

Supplementary Table 8 Effect of H_2/CO ratio on catalytic performances of K^+−ZnO−ZrO_2|H-MOR−DA−12MR for syngas conversion.

Supplementary Table 9 Effect of CO/CH_3OH ratio on catalytic performances of H-MOR−DA−12MR for methanol carbonylation with CO.

Supplementary Table 10 Catalytic performances of Cu−Zn−Al oxide, Cu−Zn−Al|H-MOR−DA−12MR and Cu−Zn−Al|H-MOR−DA−12MR|Pt−Sn/SiC for syngas conversion.

Supplementary Table 11 Catalytic performances of H-MOR−DA−12MR for carbonylation of CH_3OH in CO and syngas streams.

Supplementary Table 12 Effect of sizes of catalyst granules on catalytic performances of K^+−ZnO−ZrO_2|H-MOR−DA−12MR|Pt−Sn/SiC for syngas conversion.

Supplementary Table 13 Effect of amount of quartz wool on catalytic performances of K^+−ZnO−ZrO_2|H-MOR−DA−12MR|Pt−Sn/SiC for syngas conversion.

Supplementary Table 14 Ethanol conversion over H-MOR−DA−12MR catalyst.

Supplementary Table 15 Effect of amount of quartz wool on catalytic performances with H-MOR−DA−12MR|Pt−Sn/SiC for acetic acid conversion.

Supplementary Table 16 Effect of configuration of catalytic system composed of K^+−ZnO−ZrO_2, H-MOR−DA−12MR and Pt−Sn/SiC for syngas conversion.

Supplementary Table 17 Effect of configuration of catalytic system composed of H-MOR−DA−12MR and Pt−Sn/SiC for acetic acid conversion.

Supplementary Note 1

Supplementary Note 2

Supplementary References

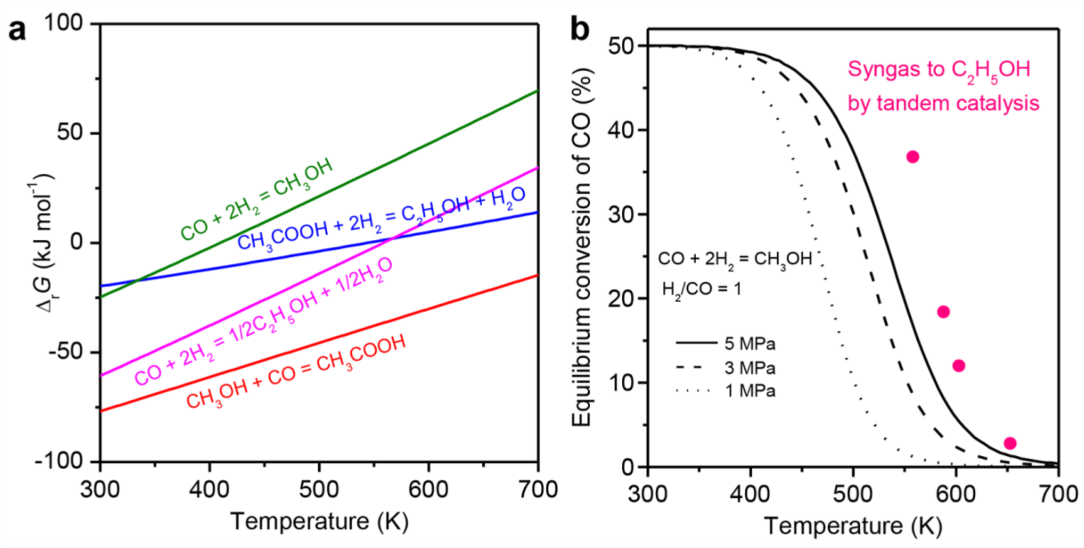

Supplementary Figure 1 Thermodynamic analyses. **a,** Gibbs free energy changes ($\Delta_r G$) for syngas to methanol, methanol carbonylation, acetic acid hydrogenation and direct conversion of syngas to ethanol. **b,** Equilibrium CO conversions for syngas to methanol and syngas to ethanol by tandem catalysis. Conditions for calculations of equilibrium conversions: H_2/CO = 1:1; syngas pressures = 1, 3 and 5 MPa. The calculation was based on HSC 5.0 chemistry software.

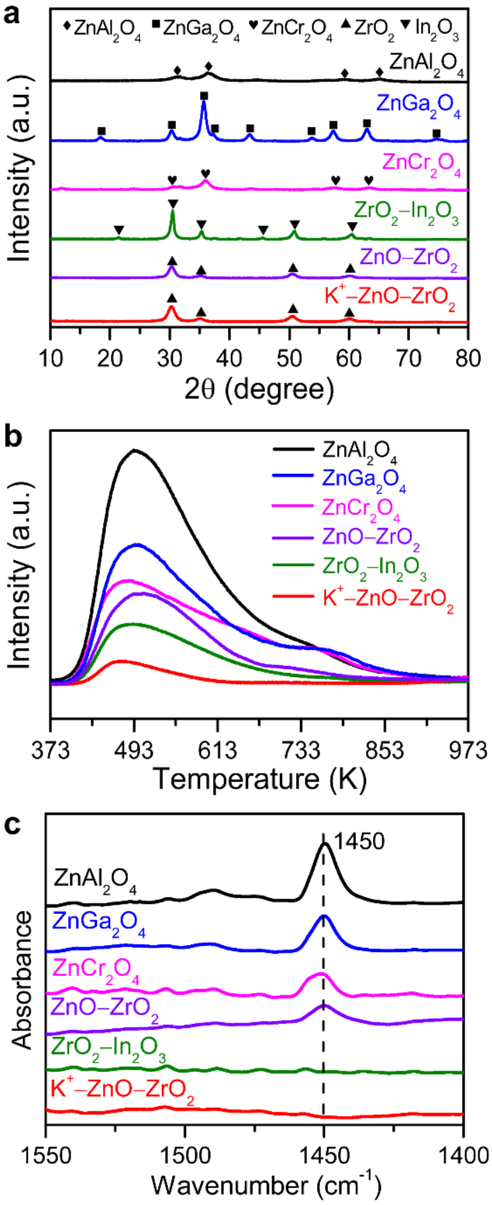

Supplementary Figure 2 Characterizations of metal oxides. **a,** XRD patterns. **b,** NH_3-TPD profiles. **c,** Pyridine-adsorbed FT-IR spectra.

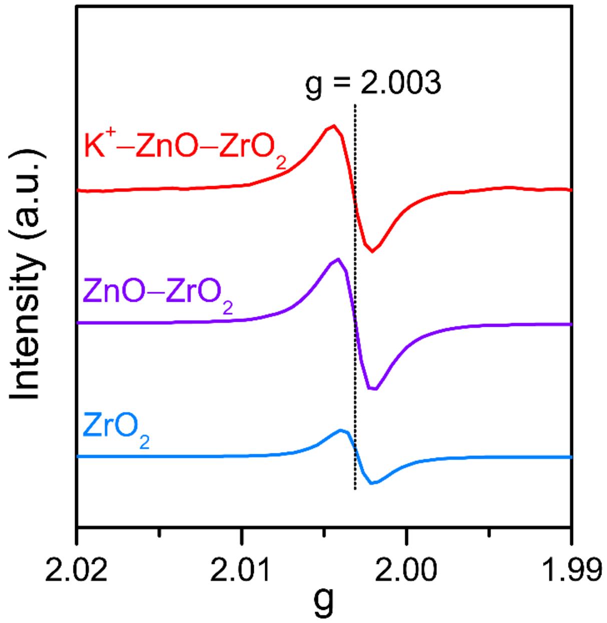

Supplementary Figure 3 EPR spectra for ZrO_2, $ZnO{-}ZrO_2$ and $K^{+}{-}ZnO{-}ZrO_2$ samples.

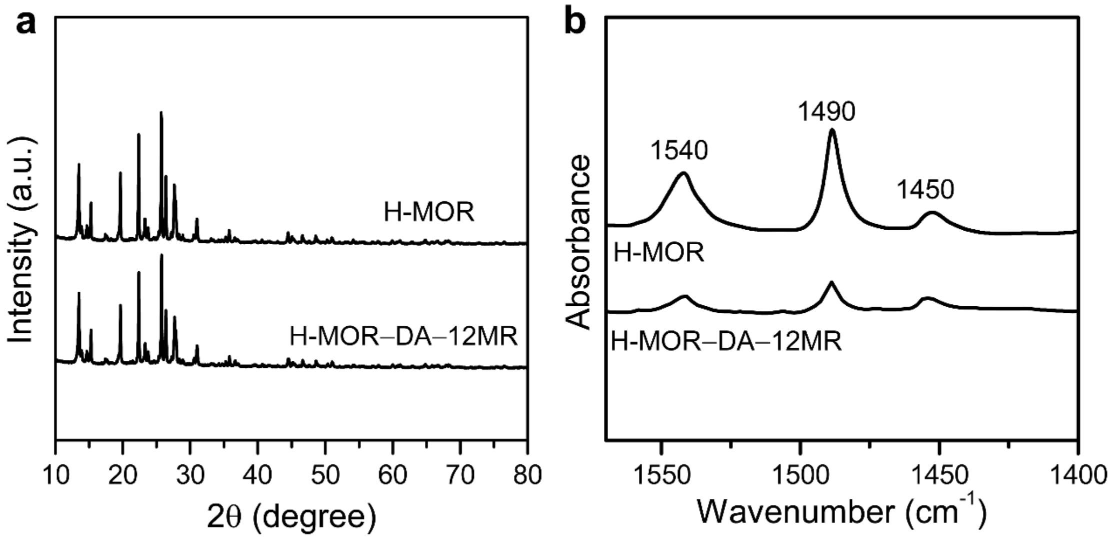

Supplementary Figure 4 Characterizations of H-MOR and H-MOR−DA−12MR zeolites. **a,** XRD patterns. **b,** Pyridine-adsorbed FT-IR spectra.

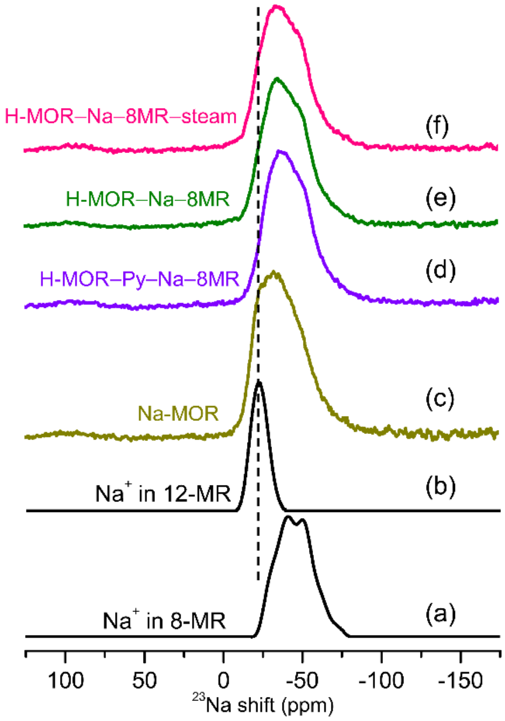

Supplementary Figure 5 ^{23}Na MAS NMR spectra for MOR zeolites. **a** and **b**, Simulation results according to the NMR parameters reported by Hunger and co-workers[1]. **c,** Conventional Na-MOR. **d,** H-MOR−Py−Na−8MR obtained by ion-exchanging of H-MOR pre-adsorbed pyridine with Na^+. **e,** H-MOR−Na−8MR obtained by calcining the H-MOR−Py−Na−8MR to remove pyridine. **f,** H-MOR−Na−8MR−steam obtained by steam treating the H-MOR−Na−8MR. The chemical shifts at -14 and -24 ppm can be assigned to the Na^+ cations in 12-MR and 8-MR channels, respectively.

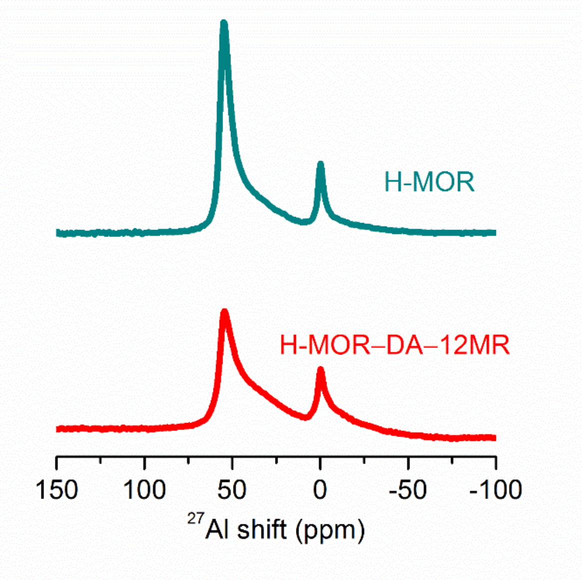

Supplementary Figure 6 ^{27}Al MAS NMR spectra for H-MOR and H-MOR−DA−12MR samples. The peaks at 54 and 0 ppm can be assigned to Al species in framework and extra-framework positions with tetrahedral and octahedral coordinations, respectively.

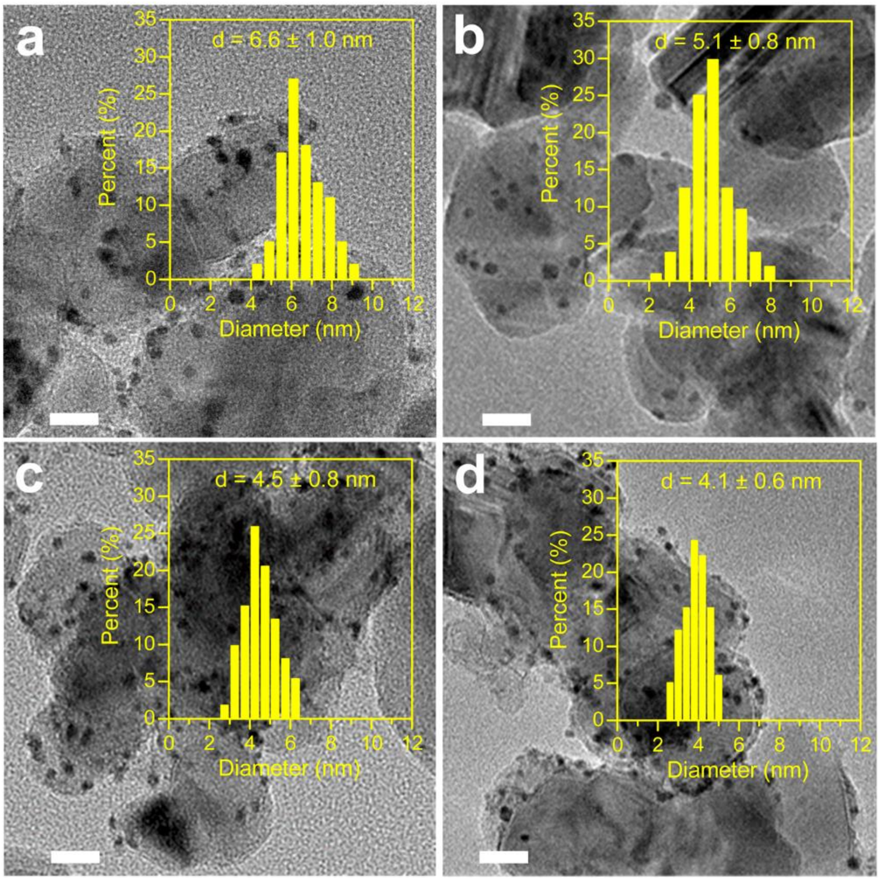

Supplementary Figure 7 TEM micrographs with particle size distributions. **a,** Pt/SiC. **b,** Pt−0.6 wt% Sn/SiC. **c,** Pt−0.9 wt% Sn/SiC. **d,** Pt−1.5 wt% Sn/SiC. The content of Pt was 1.0 wt%. Scale bar: 20 nm.

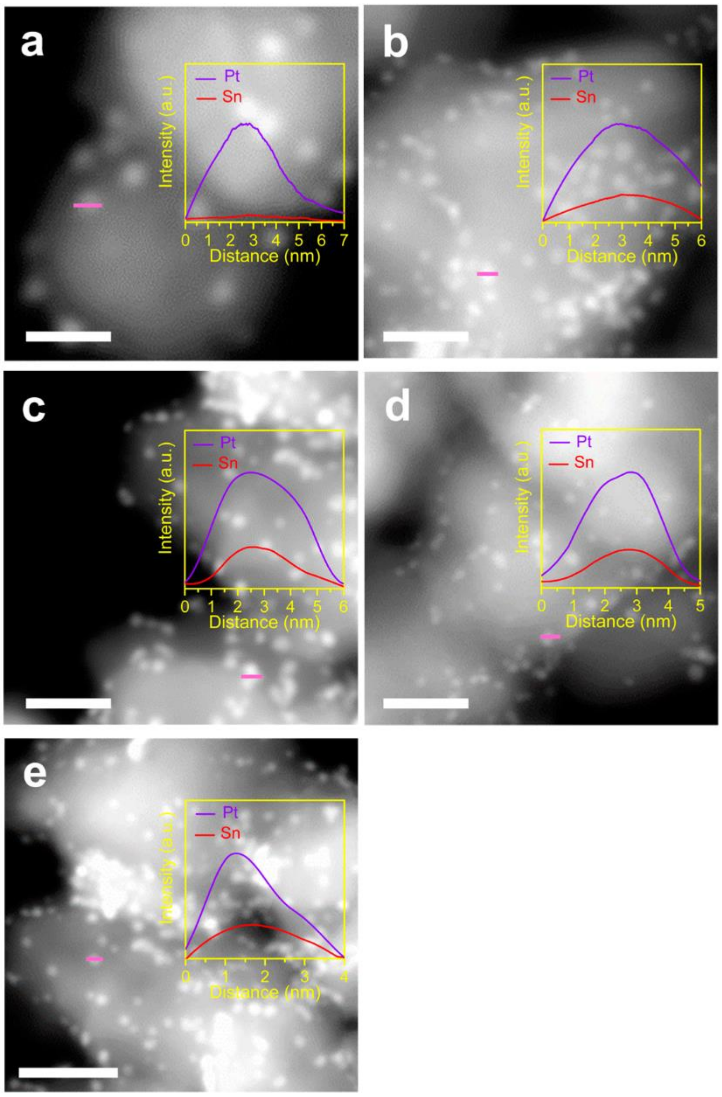

Supplementary Figure 8 Dark-field TEM micrographs with line-scan EDS analyses. The line-scan EDS was performed across one nanoparticle in the micrographs (marked with pink line). **a,** Pt/SiC. **b,** Pt−0.6 wt% Sn/SiC. **c,** Pt−0.9 wt% Sn/SiC. **d,** Pt−1.2 wt% Sn/SiC. **e,** Pt−1.5 wt% Sn/SiC. The content of Pt was 1.0 wt%. Scale bar: 20 nm.

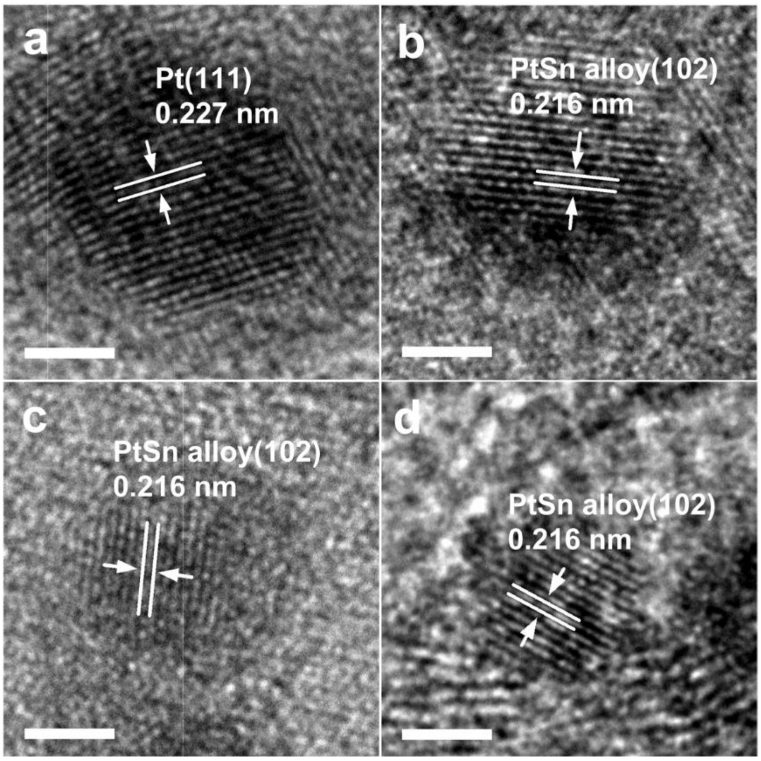

Supplementary Figure 9 HRTEM images for Pt/SiC and Pt−Sn/SiC. **a,** Pt/SiC. **b,** Pt−0.6 wt% Sn/SiC. **c,** Pt−0.9 wt% Sn/SiC. **d,** Pt−1.5 wt% Sn/SiC. The content of Pt was 1.0 wt%. Scale bar: 2 nm.

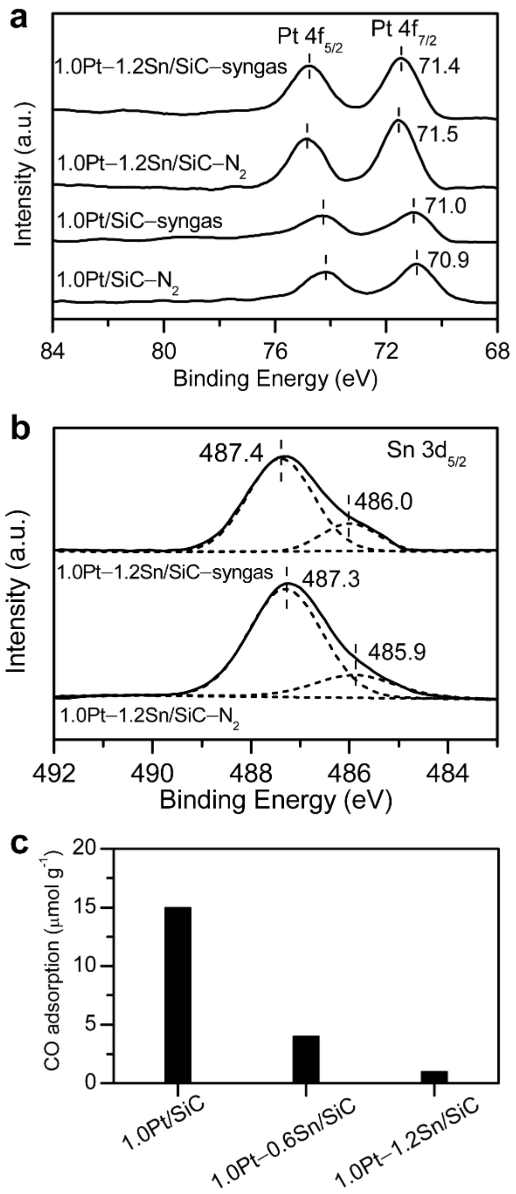

Supplementary Figure 10 XPS and CO chemisorption results for Pt/SiC and Pt−Sn/SiC. **a,** Pt 4f XPS spectra for 1.0 wt% Pt/SiC and 1.0 wt% Pt−1.2 wt% Sn/SiC after N_2 and syngas treatments. **b,** Sn 3d XPS spectra for 1.0 wt% Pt−1.2 wt% Sn/SiC after N_2 and syngas treatments. **c,** CO chemisorption amounts for 1.0 wt% Pt/SiC, 1.0 wt% Pt−0.6 wt% Sn/SiC and 1.0wt% Pt−1.2 wt% Sn/SiC.

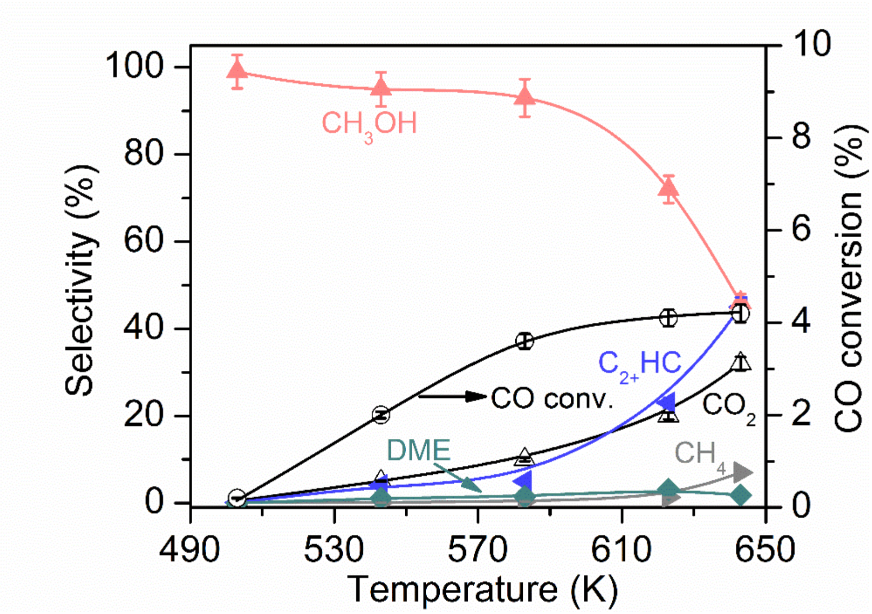

Supplementary Figure 11 Effect of temperature on syngas conversion on K^+−ZnO−ZrO_2. Reaction conditions: weight of K^+−ZnO−ZrO_2 = 0.66 g; H_2/CO = 1:1; P = 5.0 MPa; F = 25 mL min^{-1}; T = 503-643 K; time on stream, 20 h. The experiments in each case were performed for three times. The error bar represents the relative deviation, which is within 5%.

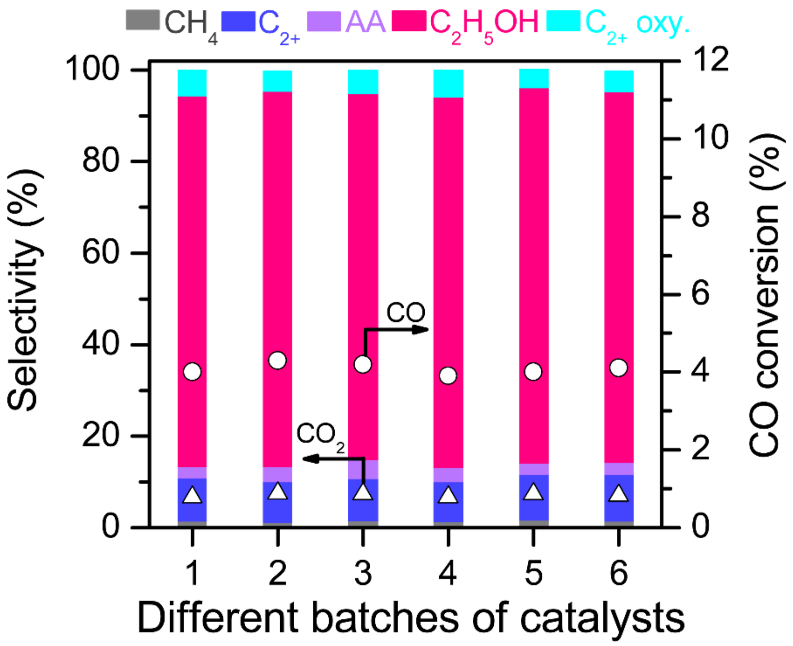

Supplementary Figure 12 Reproducibility of syngas conversion. The catalysts of the triple tandem system were from different batches. Reaction conditions: weights of K^+−ZnO−ZrO_2, H-MOR−DA−12MR and Pt−Sn/SiC = 0.66, 0.66 and 0.66 g; H_2/CO = 1:1; P = 5.0 MPa; T = 543 K; F = 25 mL min^{-1}; time on stream, 20 h.

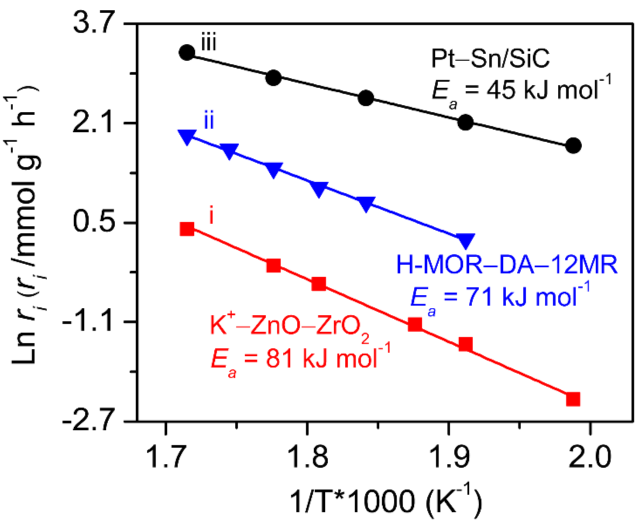

Supplementary Figure 13 Arrhenius plots. **i,** Methanol synthesis on K^+−ZnO−ZrO_2. **ii,** Methanol carbonylation on H-MOR−DA−12MR. **iii,** Acetic acid hydrogenation on Pt−Sn/SiC. Reaction conditions: **i**, weight of K^+−ZnO−ZrO_2, 0.66 g; H_2/CO = 1:1; *P* = 5.0 MPa; *F* = 25 mL min^{-1}; time on stream, 20 h. **ii**, Weight of H-MOR−DA−12MR, 0.050 g; *P* = 5.0 MPa; *F*(CH_3OH) = 1.48 mmol h^{-1}; CO/CH_3OH = 60; time on stream, 20 h. **iii**, Weight of Pt−Sn/SiC, 0.05 g; *P* = 5.0 MPa; *F*(CH_3COOH) = 10.5 mmol h^{-1}; H_2/CH_3COOH = 7.5; time on stream, 20 h.

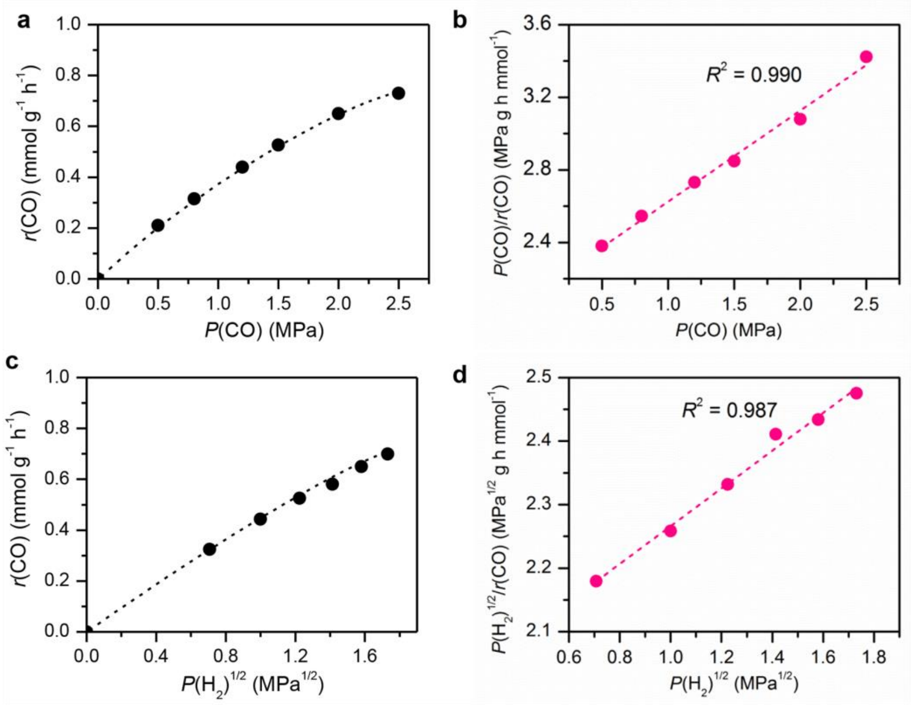

Supplementary Figure 14 Effects of partial pressures of CO and H_2. **a,** CO conversion rate, *r*(CO), versus CO partial pressure, *P*(CO). **b,** Plot of *P*(CO)/*r*(CO) versus *P*(CO). Reaction conditions: $P(H_2)$ = 2.5 MPa; *T* = 583 K; *F*(total) = 40 mL min^{-1}; *P*(total) = 5 MPa; time on stream, 20 h. **c,** *r*(CO) versus square root of H_2 partial pressure, $P(H_2)^{1/2}$. **d,** Plot of $P(H_2)^{1/2}/r(CO)$ versus $P(H_2)^{1/2}$. Reaction conditions: *P*(CO) = 2.0 MPa; *T* = 583 K; *F*(total) = 40 mL min^{-1}; *P*(total) = 5 MPa; time on stream, 20 h. In all cases, the weights of K^+−ZnO−ZrO_2, H-MOR−DA−12MR and Pt−Sn/SiC catalysts were 0.66, 0.66 and 0.66 g, respectively.

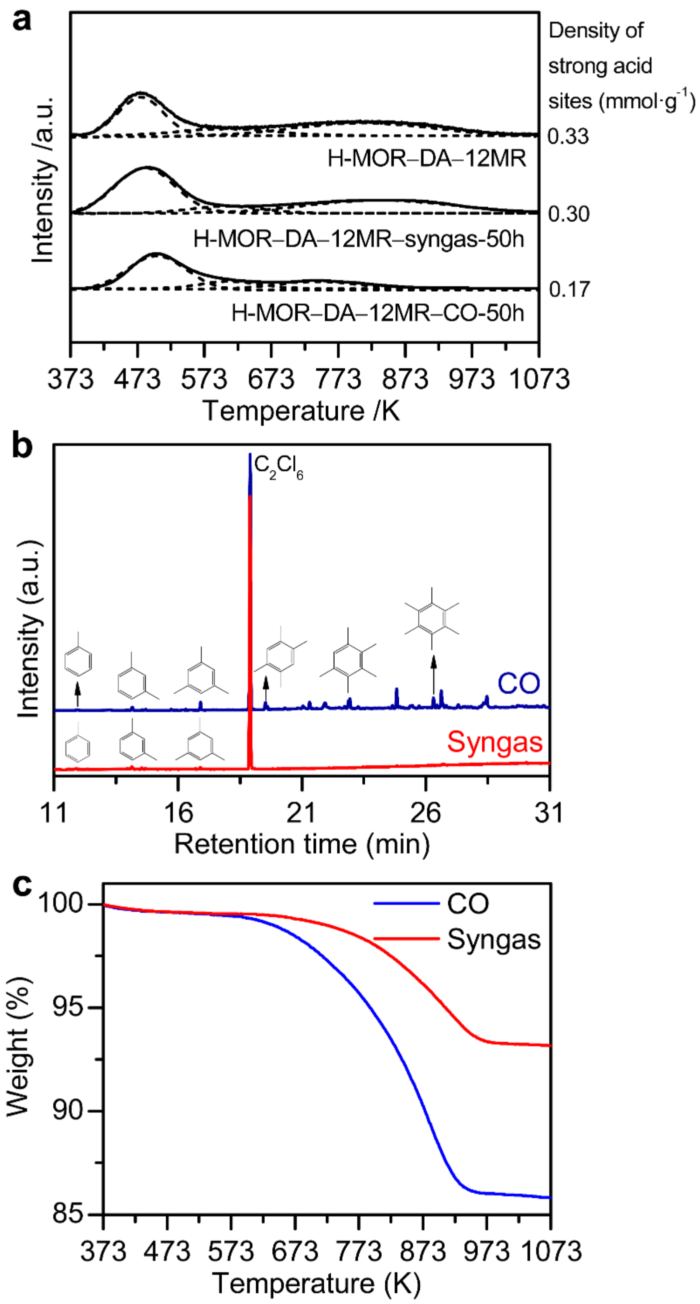

Supplementary Figure 15 Characterizations of used H-MOR and H-MOR−DA−12MR. **a,** NH_3-TPD. **b,** GC-MS. **c,** TG. Both H-MOR and H-MOR−DA−12MR were characterized after methanol carbonylation in CO and syngas streams.

Supplementary Table 1 Typical catalysts reported for the conversion of syngas to ethanol

Catalyst	*T* (K)	*P* (MPa)	H_2/CO	CO conv. (%)	CO_2 select. (%)	Selectivity (%)					Ref.
						CH_4	C_{2+} HC[a]	MeOH	EtOH	Other oxy.[b]	
1.0%Rh/(5.0%FeO_x−SiO_2)	523	2.0	2.0	6.1	2.7	22	4.9	22	46	5.1	2
2.5Rh4Fe0.5Li/γ-Al_2O_3	533	2.0	2.0	11	14	30	19	10	35	6.0	3
RhMn-in-CNTs	593	3.0	2.0	8.3	23	23	16	1.5	40	19	4
Rh−Mn/SiO_2	553	5.4	2.0	25	0	38	-	3.9	56	1.6[c]	5
Rh/$Ce_{0.8}Zr_{0.2}O_2$	548	2.4	2.0	27	10	40	2.3	8.8	39	10	6
7Rh/NFe_2O_3	513	0.1	0.5	2.0	-	31	34	2.7	27	5.1	7
$K(C)_{06}Co_{05}MoAl_{1000}$	533	7.0	1.0	4.4	33	20	12	13	47	8.2	8
$Ni_1Mo_1K_{0.05}$–Ni/CNTs	558	5.0	1.0	15	4.1	30[d]		19	24	27	9
$La_{0.7}Sr_{0.3}Co_{0.65}Ga_{0.35}$	573	4.0	2.0	9.7	7.7	43[d]		15	32	10	10
$Co_4Mn_1K_{0.3}$	513	4.0	1.5	~7.0	-	-	-	-	44[e]		11
CuCo-Red/15%CNTs	503	3.0	2.0	45	1.4	9.7	25	3.3	29	33	12
K−CuCo/MoO_x	543	4.0	1.0	< 2.0	22-42	-	-	19	26[e]		13
Co_2Cu_1	513	4.0	1.5	5.7	1.4	17	45	12	26[e]		14
S_2-CuFeMg	573	4.0	2.0	57	12	17	27	19	16	21	15
3DOM Cu_2Fe_1	533	4.8	1.0	59	45	63[d]		4.4	32[e]		16

(**a**) C_{2+} hydrocarbons. (**b**) C_{2+} alcohols and aldehydes. (**c**) C_{2+} hydrocarbons and C_{2+} oxy. (**d**) CH_4 and C_{2+} hydrocarbons. (**e**) EtOH and C_{2+} alcohols.

Supplementary Table 2 Typical catalysts reported for the conversion of syngas to acetic acid

Catalyst	*T* (K)	*P* (MPa)	H_2/CO	CO conv. (%)	Selectivity (%)				Ref.
					HC[a]	EtOH	AA[b]	Other oxy.[c]	
Rh/NaY−WA	523	1.0	0.33	0.70	28	0	68	4.2	17
Rh/NaY	503	1.0	1.0	0.44	32	0	62	6.7	18
Rh/SiO_2−CDN	593	3.0	2.0	0.30	48	5.4	33	14	19
Rh−Sm/SiO_2	553	3.0	2.0	3.4	40	15	28	17	20
Rh−Mn/SiO_2	563	3.0	2.0	3.6	43	17	18	22	21
Rh−Mn−Li−Fe/SiO_2	578	5.0	2.0	5.4	32	30	16	22	22
Rh−Mn−Fe−M/SiO_2	200 -350	2.5-12	2.5-1.5	-	40[d]		60[e]		23
MOR and solid acid catalyst[f]	508	1.4	-	-	-	-	49	-	24
K^+−ZnO−ZrO_2|H-MOR−DA−12MR	543	5.0	1.0	3.9	9.0	0	90	0.8	This work

(**a**) Hydrocarbons. (**b**) Acetic acid. (**c**) C_{2+} alcohols and aldehydes. (**d**) Weight percentage. (**e**) Including ethanol, acetic acid, and acetaldehyde by weight. (**f**) The results were from a patent[24]; the route combined carbonylation of dimethyl ether with syngas to methyl acetate and methyl acetate dehydration-hydrolysis to acetic acid and dimethyl ether over MOR and solid acid catalyst.

Supplementary Table 3 Conversion of syngas using combination of K^+−ZnO−ZrO_2|H-MOR−DA−12MR|Cu/SiO_2 at different temperatures[a]

Temp. (K)	CO conv. (%)	CO_2 select. (%)	Selectivity (%)[b]								
			CH_4	$C_{2-4}^{=}$	C_{2-4}^{0}	C_{5+}[c]	CH_3OH (DME)	EtOH	MA[d]	EA[e]	AA[f]
503	0.7	19	0.2	1.8	0.1	0	0	21	30	9.2	38
543	4.2	28	4.9	2.1	16	1.1	0	34	17	7.2	18
583	7.8	38	13	5.0	24	1.4	1.5(0)	46	2.3	5.7	1.1

(**a**) Reaction conditions: weights of K^+−ZnO−ZrO_2, H-MOR−DA−12MR, Cu/SiO_2 = 0.66, 0.66 and 0.66 g; H_2/CO = 1:1; P = 5.0 MPa; F = 25 mL min^{-1}; time on stream, 20 h. (**b**) The selectivity was calculated on a molar carbon basis. Carbon balances were all 95-99%. (**c**) C_{5+} hydrocarbons. (**d**) Methyl acetate. (**e**) Ethyl acetate. (**f**) Acetic acid.

Supplementary Table 4 Effect of Sn content in Pt−Sn/SiC on catalytic performances of K^+−ZnO−ZrO_2|H-MOR−DA−12MR|Pt−Sn/SiC for syngas conversion[a]

Sn content (wt%)	CO conv. (%)	CO_2 select. (%)	Selectivity (%)[b]								
			CH_4	$C_{2-4}^{=}$	C_{2-4}^{0}	C_{5+}[c]	CH_3OH &DME	EtOH	MA[d]	EA[e]	AA[f]
0	5.6	10	1.2	13	4.3	2.9	0.2	2.2	0.6	0.1	75
0.6	5.6	15	2.6	14	5.5	2.5	0.7	44	1.8	7.7	21
0.9	5.6	17	2.3	16	5.9	4.1	0	56	0.6	5.3	9.7
1.2	5.7	18	2.1	16	4.7	5.1	0	70	0.3	1.1	0.5
1.5	5.7	20	1.9	16	5.6	4.1	0	71	0.3	0.9	0.1

(**a**) Reaction conditions: weights of K^+−ZnO−ZrO_2, H-MOR−DA−12MR and Pt−Sn/SiC = 0.66, 0.66 and 0.66 g; H_2/CO = 1:1; P = 5.0 MPa; T = 583 K; F = 25 mL min^{-1}; time on stream, 20 h. (**b**) The selectivity was calculated on a molar carbon basis. Carbon balances were all 95-99%. (**c**) C_{5+} hydrocarbons. (**d**) Methyl acetate. (**e**) Ethyl acetate. (**f**) Acetic acid.

Supplementary Table 5 Effect of Pt loading in Pt−Sn/SiC on catalytic performances of K^+−ZnO−ZrO_2|H-MOR−DA−12MR|Pt−Sn/SiC for syngas conversion[a]

Pt loading (%)	CO conv. (%)	CO_2 select. (%)	Selectivity (%)[b]								
			CH_4	$C_{2-4}^{=}$	C_{2-4}^{0}	C_{5+}[c]	CH_3OH &DME	EtOH	MA[d]	EA[e]	AA[f]
0.5	5.7	17	1.5	14	4.4	4.6	0	68	2.5	4.2	0.7
1.0	5.7	18	2.1	16	4.7	5.1	0	70	0.3	1.1	0.5
1.2	5.8	18	2.2	12	8.1	4.1	0	70	0.8	2.8	0.3
1.5	6.6	25	2.3	8.8	13	3.5	0	70	0.1	1.6	0.3
2.0	10	35	1.2	6.7	14	5.5	0	72	0.2	0.2	0.1

(**a**) Reaction conditions: weights of K^+−ZnO−ZrO_2, H-MOR−DA−12MR and Pt−Sn/SiC = 0.66, 0.66 and 0.66 g; H_2/CO = 1:1; P = 5.0 MPa; T = 583 K; F = 25 mL min^{-1}; time on stream, 20 h. (**b**) The selectivity was calculated on a molar carbon basis. Carbon balances were all 95-99%. (**c**) C_{5+} hydrocarbons. (**d**) Methyl acetate. (**e**) Ethyl acetate. (**f**) Acetic acid.

Supplementary Table 6 Effect of ratio of amounts of three catalyst components on catalytic performances of K^+−ZnO−ZrO_2|H-MOR−DA−12MR|Pt−Sn/SiC for syngas conversion[a]

Amounts of components (g)	CO conv. (%)	CO_2 select. (%)	Selectivity (%)[b]								
			CH_4	$C_{2-4}^{=}$	C_{2-4}^{0}	C_{5+}[c]	CH_3OH &DME	EtOH	MA[d]	EA[e]	AA[f]
0.66:0.66:0.66	5.7	18	2.1	16	4.7	5.1	0	70	0.3	1.1	0.5
0.80:0.66:0.66	7.0	18	1.5	16	4.5	5.2	0	71	0.6	1.0	0.5
1.00:0.66:0.66	8.4	19	1.7	17	4.9	5.4	0	68	1.1	1.3	0.9
1.50:0.66:0.66	9.0	20	1.7	20	7.6	5.9	0	59	2.2	1.9	1.1
1.50:0.80:0.66	9.3	20	2.2	19	7.2	5.3	0	62	2.0	1.5	0.5
1.50:1.00:0.66	9.7	22	2.3	19	6.0	4.6	0	64	1.6	1.6	0.7
0.66:0.53:0.66	5.6	18	1.5	15	5.2	5.9	0.7	68	1.8	1.2	0.5
0.66:0.80:0.66	5.8	18	1.9	14	4.9	4.7	0	71	1.4	1.6	0.2
0.66:0.66:0.17	5.6	17	1.9	13	2.3	3.4	0	58	2.5	7.1	11
0.66:0.66:0.33	5.7	17	2.1	16	4.4	3.1	0	60	0.6	3.6	10
0.66:0.66:0.53	5.7	17	1.7	19	3.1	3.6	0	70	1.0	1.0	0.5

(**a**) Reaction conditions: H_2/CO = 1:1; P = 5.0 MPa; T = 583 K; F = 25 mL min^{-1}; time on stream, 20 h. (**b**) The selectivity was calculated on a molar carbon basis. Carbon balances were all 95-99%. (**c**) C_{5+} hydrocarbons. (**d**) Methyl acetate. (**e**) Ethyl acetate. (**f**) Acetic acid.

Supplementary Table 7 Densities of Brønsted acid sites in 8-MR, 12-MR and intersection between 8-MR and 12-MR estimated from ^{1}H MAS NMR and FT-IR spectra

Sample	Si/Al[a]	Total density of Brønsted acids[b] (mmol g^{-1})	Density of Brønsted acids in 8-MR channels[c] (mmol g^{-1})	Density of Brønsted acids in intersection of 8-MR and 12-MR channels[c] (mmol g^{-1})	Density of Brønsted acids in 12-MR channels[c] (mmol g^{-1})
H-MOR	13	0.51	0.19	0.09	0.23
H-MOR−DA−12MR	19	0.33	0.17	0.06	0.10

(**a**) Measured by XRF. (**b**) Measured by ^{1}H MAS NMR in Fig. 3e. (**c**) Estimated by FT-IR in Fig. 3f.

Supplementary Table 8 Effect of H_2/CO ratio on catalytic performances of K^+−ZnO−ZrO_2|H-MOR−DA−12MR for syngas conversion[a]

H_2/CO ratio	CO conv. (%)	CO_2 select. (%)	Selectivity (%)[b]						
			CH_4	$C_{2-4}^{=}$	C_{2-4}^{0}	C_{5+}[c]	CH_3OH &DME	MA[d]	AA[e]
0.25:1	2.7	6.5	1.7	2.0	2.7	0.9	0	1.0	92
0.5:1	4.1	9.7	0.9	7.9	0.4	1.5	0	0.7	89
1:1	5.6	12	1.3	8.4	1.4	2.9	0	2.3	84
2:1	7.3	19	4.1	18	5.0	2.1	0	3.7	67
3:1	7.7	20	4.4	24	6.1	3.0	0	2.5	60

(**a**) Reaction conditions: weights of K^+−ZnO−ZrO_2 and H-MOR−DA−12MR = 0.66 g and 0.66 g; P = 5.0 MPa; T = 583 K; F = 25 mL min^{-1}; time on stream, 20 h. (**b**) The selectivity was calculated on a molar carbon basis. Carbon balances were all 95-99%. (**c**) C_{5+} hydrocarbons. (**d**) Methyl acetate. (**e**) Acetic acid.

Supplementary Table 9 Effect of CO/CH_3OH ratio on catalytic performances of H-MOR−DA−12MR for methanol carbonylation with CO[a]

CO/CH_3OH	Temp. (K)	CH_3OH conv. (%)	Selectivity (%)[b]				
			DME	MA	AA	CH_4	C_{2+}HC[c]
500	503	100	0	0.6	99	0	0
400	503	100	9.2	7.7	83	0.2	0
300	503	98	13	8.2	78	0.5	0
200	503	96	24	44	32	0.2	0
60	503	94	34	45	21	0.2	0
500	583	100	0	0.5	99	0.2	0.3
200	583	100	0	0.7	98	0.3	0.4
29	583	99	0.2	6.9	90	0.1	2.4
8.5	583	100	0	13	82	0.1	3.0
5.0	583	99	0.4	17	67	0.7	15

(**a**) Reaction conditions: weight of H-MOR−DA−12MR, 0.66 g; P = 5.0 MPa; flow rate of CH_3OH, 1.48 mmol h^{-1}; time on stream, 5 h. (**b**) The selectivity was calculated on a molar carbon basis. Carbon balances were all 95-99%. (**c**) C_{2+} hydrocarbons.

Supplementary Table 10 Catalytic performances of Cu−Zn−Al oxide, Cu−Zn−Al|H-MOR−DA−12MR and Cu−Zn−Al|H-MOR−DA−12MR|Pt−Sn/SiC for syngas conversion[a]

Catalyst	CO conv. (%)	CO_2 select. (%)	Selectivity (%)[b]								
			CH_4	$C_{2-4}^{=}$	C_{2-4}^{0}	C_{5+}[c]	CH_3OH (DME)	EtOH	MA[d]	EA[e]	AA[f]
Cu−Zn−Al[g]	11	2.5	0.1	0.7	0.3	0.1	95(3.6)	0	0	0	0
Cu−Zn−Al|H-MOR−DA−12MR[g]	16	12	8.6	7.9	1.2	0.8	14(60)	0	6.1	0	1.0
Cu−Zn−Al[h]	33	39	4.9	48	6.0	4.0	30(7.0)	0	0	0	0
Cu−Zn−Al|H-MOR−DA−12MR[h]	37	37	6.3	59	12	10	0(1.0)	0	5.1	0	8.8
Cu−Zn−Al[i]	0.8	2.3	0.4	2.0	0	0.7	96(0.6)	0	0	0	0
Cu−Zn−Al[j]	1.9	2.8	0.5	3.3	2.6	1.1	92(0.6)	0	0	0	0
Cu−Zn−Al[k]	3.6	4.8	1.2	4.5	2.8	2.3	88(0.8)	0	0	0	0
Cu−Zn−Al|H-MOR−DA−12MR[i]	1.5	1.0	0.2	3.6	0.2	0.5	0	0	2.0	0	93
Cu−Zn−Al|H-MOR−DA−12MR[j]	3.7	3.4	0.4	8.4	4.3	6.3	0	0	1.7	0	79
Cu−Zn−Al|H-MOR−DA−12MR[k]	5.2	6.2	0.6	11	7.2	9.5	0	0	3.0	0	69
Cu−Zn−Al|H-MOR−DA−12MR|Pt−Sn/SiC[i]	1.5	2.5	0.3	1.2	0.2	1.8	7.4(0)	81	2.4	6.1	0
Cu−Zn−Al|H-MOR−DA−12MR|Pt−Sn/SiC[j]	3.8	3.2	0.4	9.1	4.5	6.0	2.0(0)	71	1.6	4.9	0.7
Cu−Zn−Al|H-MOR−DA−12MR|Pt−Sn/SiC[k]	5.5	8.0	0.9	12	8.2	10	0.6(0)	60	3.5	4.2	0.7

(**a**) Reaction conditions: weights of Cu−Zn−Al, H-MOR−DA−12MR and Pt−Sn/SiC = 0.05 (mixed with silica sand), 0.66 and 0.66 g; H_2/CO = 1:1; P = 5.0 MPa; F = 25 mL min^{-1}; time on stream, 20 h. (**b**) The selectivity was calculated on a molar carbon basis. Carbon balances were all 95-99%. (**c**) C_{5+} hydrocarbons. (**d**) Methyl acetate. (**e**) Ethyl acetate. (**f**) Acetic acid. (**g**) Weight of Cu−Zn−Al, 0.66 g; T = 503 K. (**h**) Weight of Cu−Zn−Al, 0.66 g; T = 583 K. (**i**) T = 503 K. (**j**) T = 523 K. (**k**) T = 543 K.

Supplementary Table 11 Catalytic performances of H-MOR−DA−12MR for carbonylation of CH_3OH in CO and syngas streams[a,b]

Reactants	CH_3OH conv. (%)	Selectivity (%)				
		DME	MA[c]	AA[d]	CH_4	C_{2+}HC [e]
CH_3OH + CO	99	0	2.0	89	0.1	8.6
CH_3OH + Syngas	100	0	1.2	90	0.6	8.0

(**a**) Reaction conditions in CO: W = 1.0 g; P = 5.0 MPa; T = 583 K; $F(CH_3OH)$ = 1.48 mmol h^{-1}; F(95%CO-5%Ar) = 12 mL min^{-1}; time on stream, 3 h. (**b**) Reaction conditions in syngas: W = 1.0 g; P = 5.0 MPa; T = 583 K; $F(CH_3OH)$ = 1.48 mmol h^{-1}; F(48%CO-48%H_2-4%Ar) = 24 mL min^{-1}; time on stream, 3 h. (**c**) Methyl acetate. (**d**) Acetic acid. (**e**) C_{2+} hydrocarbons.

Supplementary Table 12 Effect of sizes of catalyst granules on catalytic performances of K^+−ZnO−ZrO_2|H-MOR−DA−12MR|Pt−Sn/SiC for syngas conversion[a]

Granule size (μm)	CO conv. (%)	CO_2 select. (%)	Selectivity[b] (%)							
			CH_4	$C_{2-4}^{=}$	C_{2-4}^{0}	C_{5+}[c]	EtOH	MA[d]	EA[e]	AA[f]
600-850	5.6	17	1.9	15	4.4	4.0	70	1.2	2.0	1.0
250-600	5.7	18	2.1	16	4.7	5.1	70	0.3	1.1	0.5
180-250	5.9	20	1.5	20	4.2	4.4	68	0.2	1.5	0.7
125-180	6.0	21	1.6	20	4.3	4.6	68	0.3	1.0	0.5

(**a**) Reaction conditions: weights of K^+−ZnO−ZrO_2, H-MOR−DA−12MR and Pt−Sn/SiC = 0.66, 0.66 and 0.66 g; H_2/CO = 1:1; P = 5.0 MPa; T = 583 K; F = 25 mL min^{-1}; time on stream, 20 h. (**b**) The selectivity was calculated on a molar carbon basis. Carbon balances were all 95-99%. (**c**) C_{5+} hydrocarbons. (**d**) Methyl acetate. (**e**) Ethyl acetate. (**f**) Acetic acid.

Supplementary Table 13 Effect of amount of quartz wool on catalytic performances of K^+−ZnO−ZrO_2|H-MOR−DA−12MR|Pt−Sn/SiC for syngas conversion[a]

Weight of quartz wool (g)	Height of quartz wool (mm)	CO conv. (%)	CO_2 select. (%)	Selectivity (%)[b]							
				CH_4	$C_{2-4}^=$ (C_2H_4)	C_{2-4}^0	C_{5+}[c]	EtOH	MA[d]	EA[e]	AA[f]
0.060	6.2	5.7	20	1.4	15 (4.1)	4.3	3.9	71	1.0	2.6	0.8
0.045	4.7	5.7	20	1.6	16 (5.4)	4.1	3.4	70	1.0	2.9	0.8
0.030	3.1	5.7	18	2.1	16 (5.3)	4.7	5.1	70	0.3	1.1	0.5
0.015	1.6	5.7	21	1.5	18 (12)	3.8	2.3	66	3.8	3.6	1.0
0.008	0.9	5.9	26	1.6	31 (21)	6.2	2.5	52	3.0	3.0	0.5
0	0	6.1	32	1.7	45 (34)	5.5	3.3	39	2.7	1.9	1.0

(**a**) Reaction conditions: weights of K^+−ZnO−ZrO_2, H-MOR−DA−12MR and Pt−Sn/SiC = 0.66, 0.66 and 0.66 g; H_2/CO = 1:1; P = 5.0 MPa; T = 583 K; F = 25 mL min^{-1}; time on stream, 20 h. (**b**) The selectivity was calculated on a molar carbon basis. Carbon balances were all 95-99%. (**c**) C_{5+} hydrocarbons. (**d**) Methyl acetate. (**e**) Ethyl acetate. (**f**) Acetic acid.

Supplementary Table 14 Ethanol conversion over H-MOR−DA−12MR catalyst[a]

Catalyst	C_2H_5OH conv. (%)	Selectivity (%)[b]	
		C_2H_4	C_2H_6
H-MOR−DA−12MR	100	97	3.0

(**a**) Reaction conditions: weight of H-MOR−DA−12MR = 0.66 g; N_2; P = 5.0 MPa; T = 583 K; $F(N_2)$ = 25 mL min^{-1}; $F(C_2H_5OH)$ = 1.05 mmol h^{-1}; time on stream, 8 h. (**b**) The selectivity was calculated on a molar carbon basis.

Supplementary Table 15 Effect of amount of quartz wool on catalytic performances with H-MOR−DA−12MR|Pt−Sn/SiC for acetic acid conversion[a]

Weight of quartz wool (g)	Height of quartz wool (mm)	CH_3COOH conv. (%)	Selectivity (%)[b]							
			CH_4	$C_{2-4}^{=}$ (C_2H_4)	C_{2-4}^{0}	C_{5+}[c]	EtOH	MA[d]	EA[e]	CO_x[f]
0.060	6.2	99	0.1	5.3(1.4)	1.2	0.8	80	4.0	5.6	2.9
0.045	4.7	99	0.1	6.2(1.9)	1.3	0.7	79	4.1	5.8	2.8
0.030	3.1	99	0.2	7.2(2.1)	1.4	0.6	78	4.2	5.9	2.5
0.015	1.6	98	0.2	19(13)	1.9	0.7	66	4.6	4.5	2.7
0.008	0.9	98	0.1	29(24)	2.1	0.5	58	4.0	3.9	2.7
0	0	97	0.1	48(44)	2.9	0.6	38	4.1	3.6	2.5

(**a**) Reaction conditions: weights of H-MOR−DA−12MR and Pt−Sn/SiC = 0.66 and 0.66 g; H_2/CO = 1/1; P = 5.0 MPa; T = 583 K; F = 25 mL min^{-1}; F(AA) = 1.05 mmol h^{-1}; time on stream, 8 h. (**b**) The selectivity was calculated on a molar carbon basis. (**c**) C_{5+} hydrocarbons. (**d**) Methyl acetate. (**e**) Ethyl acetate. (**f**) CO and CO_2.

Supplementary Table 16 Effect of configuration of catalytic system composed of K^+−ZnO−ZrO_2, H-MOR−DA−12MR and Pt−Sn/SiC for syngas conversion[a]

Configuration	CO conv. (%)	CO_2 select. (%)	Selectivity (%)[b]					
			CH_4	$C_{2-4}^{=}$ (C_2H_4)	C_{2-4}^{0} (C_2H_6)	C_{5+}[c]	EtOH	Other oxy.[d]
Layer-by-layer[e]	5.7	18	2.1	16(5.3)	4.7(2.3)	5.1	70	1.9
Granule mixing	6.7	24	2.4	80(55)	14(11)	4.2	0	0
Grinding mixing	6.9	26	2.0	82(68)	12(11)	4.0	0	0

(**a**) Reaction conditions: weights of K^+−ZnO−ZrO_2, H-MOR−DA−12MR and Pt−Sn/SiC = 0.66, 0.66 and 0.66 g; H_2/CO = 1:1; P = 5.0 MPa; T = 583 K; F = 25 mL min^{-1}; time on stream, 20 h. (**b**) The selectivity was calculated on a molar carbon basis. Carbon balances were all 95-99%. (**c**) C_{5+} hydrocarbons. (**d**) Methyl acetate, ethyl acetate and acetic acid. (**e**) Separated by quartz wool.

Supplementary Table 17 Effect of configuration of catalytic system composed of H-MOR−DA−12MR and Pt−Sn/SiC for acetic acid conversion[a]

Configuration	CH_3COOH conv. (%)	Selectivity (%)[b]							
		CH_4	$C_{2-4}^{=}$ (C_2H_4)	C_{2-4}^{0} (C_2H_6)	C_{5+}[c]	EtOH	MA[d]	EA[e]	CO_x[f]
Layer-by-layer[g]	99	0.2	7.2(2.1)	1.4(0.6)	0.6	82	3.2	2.9	2.5
Granule mixing	99	0.3	78(58)	18(14)	0.5	0	0	0	3.2
Grinding mixing	99	0.1	82(70)	14(12)	0.3	0	0	0	3.6

(**a**) Reaction conditions: weights of H-MOR−DA−12MR and Pt−Sn/SiC = 0.66 and 0.66 g; H_2/CO = 1:1; P = 5.0 MPa; T = 583 K; F = 25 mL min^{-1}; F(AA) = 1.05 mmol h^{-1}; time on stream, 8 h. (**b**) The selectivity was calculated on a molar carbon basis. Carbon balances were all 95-99%. (**c**) C_{5+} hydrocarbons. (**d**) Methyl acetate. (**e**) Ethyl acetate. (**f**) CO and CO_2. (**g**) Separated by quartz wool.

Supplementary Note 1

Explanation of the thermodynamic calculations in Supplementary Figure 1: The tandem catalysis is composed of three steps, i.e., methanol synthesis, methanol carbonylation and acetic acid hydrogenation, and the three catalyst components are separated with each other by quartz wool in one fixed-bed flow reactor. The reaction in each step occurs relatively independently over the corresponding catalyst bed in the reactor and the intermediates, i.e., methanol and acetic acid, cannot be mixed together. Thus, the thermodynamics of each step should be considered. Therefore, the thermodynamics of tandem catalysis is different from that of the direct conversion of syngas to ethanol with reactants, reaction intermediates and products mixed together, which is thermodynamically feasible at ≤ 600 K (Supplementary Figure 1a, pink line). It is clear that the tandem catalysis is thermodynamically limited by the first step due to its large positive $\Delta_r G$ values at 500-600 K, the temperatures used in this work. The equilibrium CO conversion for syngas to ethanol by tandem catalysis was calculated by a simplified $CO + 2H_2 \rightarrow CH_3OH$, $CH_3OH + CO \rightarrow CH_3COOH$ and $CH_3COOH + 2H_2 \rightarrow C_2H_5OH + H_2O$ reaction route. The initial ratio of H_2/CO and reaction pressure were 1:1 and 5 MPa, respectively. The equilibrium CO conversions at four temperatures were calculated for syngas to ethanol by tandem catalysis.

Supplementary Note 2

Illustration of the kinetic results in Supplementary Figure 14: According to the Langmuir-Hinshelwood kinetic model for the reaction between adsorbed CO and dissociatively adsorbed H species, the reaction rate can be expressed as:

$$r(\mathrm{CO}) = k \frac{K(\mathrm{CO})P(\mathrm{CO})}{[1 + K(\mathrm{CO})P(\mathrm{CO})]} \frac{K(\mathrm{H_2})^{1/2}\,P(\mathrm{H_2})^{1/2}}{[1 + K(\mathrm{H_2})^{1/2}P(\mathrm{H_2})^{1/2}]}$$

where k and K are temperature-dependent constants. Therefore, at a fixed H_2 partial pressure, there is linear relationship between $P(\mathrm{CO})/r(\mathrm{CO})$ and $P(\mathrm{CO})$. Similarly, there is linear relationship between $P(\mathrm{H_2})^{1/2}/r(\mathrm{CO})$ and $P(\mathrm{H_2})^{1/2}$ at a fixed CO partial pressure.

Supplementary References

1. Hunger, M., Sarv, P. & Samoson, A. Two-dimensional triple-quantum ^{23}Na MAS NMR spectroscopy of sodium cations in dehydrated zeolites. *Solid State Nucl. Magn. Reson.* **9**, 115-120 (1997).
2. Wang, J., Zhang, Q. & Wang, Y. Rh-catalyzed syngas conversion to ethanol: studies on the promoting effect of FeO_x. *Catal. Today* **171**, 257-265 (2011).
3. Chen, Y., Zhang, H., Ma, H., Qian, W., Jin, F. & Ying, W. Direct conversion of syngas to ethanol over Rh-Fe/γ-Al_2O_3 catalyst: promotion effect of Li. *Catal. Lett.* **148**, 691-698 (2018).
4. Pan, X., Fan, Z., Chen, W., Ding, Y., Luo, H. & Bao, X. Enhanced ethanol production inside carbon-nanotube reactors containing catalytic particles. *Nat. Mater.* **6**, 507-511 (2007).
5. Hu, J., Wang, Y., Cao, C., Elliott, D. C., Stevens, D. J. & White, J. F. Conversion of biomass-derived syngas to alcohols and C_2 oxygenates using supported Rh catalysts in a microchannel reactor. *Catal. Today* **120**, 90-95 (2007).
6. Liu, Y., Murata, K., Inaba, M., Takahara, I. & Okabe, K. Synthesis of ethanol from syngas over Rh/$Ce_{1-x}Zr_xO_2$ catalysts. *Catal. Today* **164**, 308-314 (2011).
7. Carrillo, P., Shi, R., Teeluck, K., Senanayake, S. D. & White, M. G. *In situ* formation of FeRh nanoalloys for oxygenate synthesis. *ACS Catal.* **8**, 7279-7286 (2018).
8. Toyoda, T., Minami, T. & Qian, E. W. Mixed alcohol synthesis over sulfided molybdenum-based catalysts. *Energy Fuels* **27**, 3769-3777 (2013).
9. Ma, C., Li, H., Lin, G. & Zhang, H. Ni-decorated carbon nanotube-promoted Ni-Mo-K catalyst for highly efficient synthesis of higher alcohols from syngas. *Appl. Catal. B: Environ.* **100**, 245-253 (2010).
10. Guo, S., Li, S., Zhong, H., Gong, D., Wang, J., Kang, N., Zhang, L., Liu, G. & Liu, Y. Mixed oxides confined and tailored cobalt nanocatalyst for direct ethanol synthesis from syngas: a catalyst designing by using perovskite-type oxide as the precursor. *Ind. Eng. Chem. Res.* **57**, 2404-2415 (2018).
11. Xiang, Y. & Kruse, N. Tuning the catalytic CO hydrogenation to straight- and long-chain aldehydes/alcohols and olefins/paraffins. *Nat. Commun.* **7**, 13058 (2016).
12. Cao, A., Liu, G., Wang, L., Liu, J., Yue, Y., Zhang, L. & Liu, Y. Growing layered double hydroxides on CNTs and their catalytic performance for higher alcohol synthesis from syngas. *J. Mater. Sci.* **51**, 5216-5231 (2016).
13. Prieto, G., Beijer, S., Smith, M. L., He, M., Au, Y., Wang, Z., Bruce, D. A., de Jong, K. P., Spivey, J. J. & de Jongh, P. E. Design and synthesis of copper-cobalt catalysts for the selective conversion of synthesis gas to ethanol and higher alcohols. *Angew. Chem. Int. Ed.* **53**, 6397-6401 (2014).
14. Xiang, Y., Barbosa, R. & Kruse, N. Higher alcohols through CO hydrogenation over CoCu catalysts: influence of precursor activation. *ACS Catal.* **4**, 2792-2800 (2014).
15. Gao, W., Zhao, Y., Liu, J., Huang, Q., He, S., Li, C., Zhao, J. & Wei, M. Catalytic conversion

of syngas to mixed alcohols over CuFe-based catalysts derived from layered double hydroxides. *Catal. Sci. Technol.* **3**, 1324-1332 (2013).

16. Lu, Y., Zhang, R., Cao, B., Ge, B., Tao, F. F., Shan, J., Nguyen, L., Bao, Z., Wu, T., Pote, J. W., Wang, B. & Yu, F. Elucidating the Copper-Hägg Iron Carbide Synergistic Interactions for Selective CO Hydrogenation to Higher Alcohols. *ACS Catal.* **7**, 5500-5512 (2017).
17. Xu, B. & Sachtler, W. M. H. Rh/NaY: a selective catalyst for direct synthesis of acetic acid from syngas. *J. Catal.* **180**, 194-206 (1998).
18. Xu, B., Sun, K., Zhu, Q. & Sachtler, W. M. H. Unusual selectivity of oxygenate synthesis Formation of acetic acid from syngas over unpromoted Rh in NaY zeolite. *Catal. Today* **63**, 453-460 (2000).
19. Chen, W., Ding, Y., Jiang, D., Wang, T. & Luo, H. A selective synthesis of acetic acid from syngas over a novel Rh nanoparticles/nanosized SiO_2 catalysts. *Catal. Commun.* **7**, 559-562 (2006).
20. Luo, H., Zhang, W., Zhou, H., Huang, S., Lin, P., Ding, Y. & Lin, L. A study of Rh-Sm-V/SiO_2 catalysts for the preparation of C_2-oxygenates from syngas. *Appl. Catal. A: Gen.* **214**, 161-166 (2001).
21. Luo, H., Lin, P., Xie, S., Zhou, H., Xu, C., Huang, S., Lin, L., Liang, D., Yin, P. & Xin, Q. The role of Mn and Li promoters in supported rhodium catalysts in the formation of acetic acid and acetaldehyde. *J. Mol. Catal. A: Chem.* **122**, 115-123 (1997).
22. Yin, H., Ding, Y., Luo, H., Yan, L., Wang, T. & Lin, L. The Performance of C_2 oxygenates synthesis from syngas over Rh-Mn-Li-Fe/SiO_2 catalysts with various Rh loadings. *Energy Fuels* **17**, 1401-1406 (2003).
23. (**a**) Atkins, M. P. Process for the conversion of synthesis gas to oxygenate. *U. S. Patent*, No.: US7939571B2. (**b**) Atkins, M. P. Process for the conversion of synthesis gas to oxygenate. *U. S. Patent*, No.: US8063110B2.
24. Bristow, T. C. Integrated process for making acetic acid from syngas. *Eur. Patent*, No.: EP2935184B1.
